# Treatment of Diaphyseal Tibial Non-unions After Open Fracture: A Retrospective Observational Study on Characteristics and Outcomes

**DOI:** 10.1007/s43465-024-01235-y

**Published:** 2024-08-18

**Authors:** R. Strain, P. J. Harwood, N. K. Kanakaris, P. V. Giannoudis

**Affiliations:** 1 Academic Department of Trauma and Orthopaedics, School of Medicine, University of Leeds, Leeds, UK; 2 NIHR Leeds Biomedical Research Center, Chapel Allerton Hospital, Leeds, UK

**Keywords:** Trauma, Non-union, Fracture, Complications, Tibia, Management, Outcomes, Lower limb, Open fracture, Fracture healing

## Abstract

**Purpose:**

Non-union is a significant complication following open diaphyseal tibia fractures. Management can be complex and unpredictable. Several principles must be addressed often in combination to achieve union. The aim of this study is to report on the characteristics, management and eventual outcome of non-united open tibial fractures over a 12-year period from a level I trauma centre.

**Methods:**

This is a retrospective observational study of all adults (age 18 years and older) presenting to a level 1 trauma centre with a diaphyseal tibia fracture. Non-union was diagnosed using the standard FDA definition of incomplete union by 9 months or no progress to union in the preceding 3 months. Injury and patient demographics in addition to all interventions and complications were recorded for each patient.

**Results:**

Forty three cases of diaphyseal non-union were identified from 2008 to 2019. Only the presence of peripheral vascular disease demonstrated a statistically significant association with the development of non-union. In 44% of cases, more than one additional operation was required to achieve union. Successful union was achieved in 90% of cases with 74% of patients returning to full pre-injury function without complication.

**Conclusion:**

Management of non-union is a complex problem which requires a multifaceted and bespoke approach. We have included an algorithm to help guide decision making based on our institutional experience. A satisfactory result is achievable in the majority of patients.

## Introduction

The most agreed upon standard definition of non-union made by the FDA is a fracture that persists for a minimum of nine months or without signs of healing for three consecutive months [1]. All fractures are at risk of this complication, but there is a particular concern regarding tibial fractures, with non-union occurring 3–5 times more often than other fractures [2]. Many theories have been postulated and most implicate the tibia’s unique anatomy and subcutaneous border as a major contributing factor. This results in a suboptimal soft tissue envelope and tenuous blood supply. Unfortunately, this also leads to high rate of open fracture [3, 4] leading to more soft tissue stripping and further compounding the tibias disadvantage for union. Managing non-union in this context becomes challenging, unpredictable and expensive. The estimated cost of managing non-union in the UK is £16,330, based on a ‘best case’ scenario whereby management followed an uncomplicated course with optimal and timely recovery and no additional complications [5, 6]. Based on the current UK population of 67 million, it has been estimated that the annual bill for managing non-union could reach £320 million [6]. Unfortunately, there is no standard management of established non-union, as each case is unique with multiple causative factors. The complex interplay of these is succinctly summarised by the ‘diamond concept’ proposed by Giannoudis in 2007 [7]. The concept advocates fulfilling certain criteria to achieve successful fracture union. These criteria include availability of osteoinductive mediators, osteogenic cells, an osteoconductive scaffold and an optimum mechanical environment. In addition, adequate vascularity around the fracture site and optimisation of host factors, for example smoking status, are also considered. The concept aims to describe an optimum situation for union but can also provide a framework to guide the management of non-union and has shown success when all elements of the diamond are addressed at the time of non-union management [8]. In the herein study, the aim is to describe the demographics and injury characteristics of patients who develop non-union following open tibial fractures to elucidate any significant risk factors from our cohort. Furthermore, the operative management and eventual outcome including complications of non-united open tibial fractures will be described over a 12-year period from a level I trauma centre.

## Materials and Methods

A retrospective observational study was conducted from our prospectively documented database of all patients 18 years and over presenting with an open diaphyseal tibia fracture (AO/OTA type 42) over a 12-year period (2008–2019). This included both isolated injuries and patients with multiple injuries. A minimum of 12 months follow up, or satisfactory discharge by the treating surgeon was required. Exclusion criteria were all closed tibial fractures, patients younger than 18 years and patients with insufficient follow up data.

Patient demographics, injury details, management and complications were recorded. Comorbidities were also documented including the Charlson comorbidity index (CCI) [9].

Non-union was diagnosed according to the FDA definition of incomplete union by 9 months or no progress to union in the preceding 3 months. A RUST score [10] was calculated for all post-operative radiographs with a score of 9 or greater and bridging callous on at least three out of four cortices representative of union. The first procedural attempt to address non-union was recorded for each patient. Surgical interventions to address non-union including use of biological augmentation to stimulate osteogenesis and enhance the local healing response were recorded for each patient. Biological stimulation used were bone marrow aspirate concentrate (BMAC), platelet rich plasma (PrP) and bone morphogenic protein 2 (BMP-2) into the fracture site or autologous bone graft (ABG). Revision of fixation was carried out as was necessary (presence of metal work failure, or signs of loosening of the in-situ implant). If no further interventions to achieve union were recorded, then this was deemed successful. Additional interventions were recorded and analysed until union was achieved. Such complications were documented as infection, failure of metal work, malunion, compartment syndrome, DVT, PE and amputation.

Statistical analysis was carried out using IBM statistical programme for the social sciences (SPSS v. 27). Nominal variables were subject to Pearson chi square test for independence or Fischer’s exact test when expected cell counts in the contingency table were less than five. Continuous variables were subject to normality testing using a Shapiro–Wilk test. An independent samples *t*-test or Mann–Whitney-*U* test was used depending on the distribution of data. A significance level of 0.05 was used for all statistical tests.

## Results

A total of 395 open diaphyseal tibia fractures were identified from 2008 to 2019. Seventeen patients were excluded due to insufficient follow up leaving 368 open tibial fractures. Non-union occurred in 43 cases (11.7%). The mean follow up time was 607 days. A comparison of the patient characteristics of both united and non-united fractures can be found in Table 1. A diagnosis of peripheral vascular disease (PVD) showed a significant association with the development of non-union. In addition, there was a trend towards higher rates of non-union in patients with concurrent diabetes or heart failure, however statistical significance was not achieved.

**Table 1:** Comparison of patient demographics and comorbidities in open tibial shaft fractures

		Union	Non-union	Significance value
Gender	Female	94 (28.9%)	10 (23.3%)	*x*^2^(1) = 0.602, *p* = 0.438
	Male	231 (71.1%)	33 (73.7%)	
Age (mean)		Med 43 (IQR 24, Range 68)	47.5 (IQR 31, Range 50)	*U* = 7815, *p* = 0.207
Smoking (*n* = 304)	No	146 (54.9%)	23 (60.5%)	*x*^2^(1) = 0.428, *p* = 0.513
	Yes	120 (45.1%)	15 (39.5%)	
Body mass index (*n* = 163)		Med 26.4 (IQR 6.95, Range 39.2)	Med 26.5 (IQR7.93, Range 18.9)	*U* = 1450.5, *p* = 0.917
Diabetes	No	304 (93.8%)	38 (88.4%)	*x*^2^(1) = 0.1780, *p* = 0.182
	Yes	20 (6.2%)	5 (11.6%)	
Peripheral vascular disease	No	322 (99.7%)	41 (95.3%)	Fisher’s exact, *p* = 0.038
	Yes	1 (0.3%)	2 (4.7%)	
COPD	No	311 (96.3%)	43 (100%)	Fisher’s exact, *p* = 0.374
	Yes	12 (3.7%)	0 (0%)	
Chronic kidney disease	No	322 (99.7%)	43 (100%)	Fisher’s exact, *p* = 1
	Yes	1 (0.3%)	0 (0%)	
Dementia	No	314 (97.2%)	41 (95.3%)	Fisher’s exact, *p* = 0.626
	Yes	9 (2.8%)	2 (4.7%)	
Heart failure	No	318 (98.5%)	41 (95.3%)	Fisher’s exact, *p* = 0.193
	Yes	5 (1.5%)	2 (4.7%)	
Charlson score		Med 0 (IQR 1, Range 11)	Med 0 (IQR 3, Range 6)	*U* = 7888.5, *p* = 0.112

*COPD* chronic obstructive pulmonary disease

Open tibial fractures occurred in isolation in 235 (59.5%) patients. In the remainder of patients, upper limb injuries were the most commonly associated injury (*n* = 59). This was followed by chest injuries (*n* = 49), pelvic injuries (*n* = 43) and spinal injuries (*n* = 36). A total of 23 patients sustained bilateral tibia fractures.

A comparison of injury specific details between united and non-united fractures can be found in Table 2. No single-injury detail showed a significant correlation with the development of non-union. However, a lower rate of non-union was observed in patients with a concomitant head injury (2.3% vs. 12.5%, *p* = 0.153), and a higher rate in patients with Gustillo-Anderson grade-3 injuries.

**Table 2:** Comparison of injury details in patients sustaining open diaphyseal tibia fractures

		Union (*n*, %)	Non-union (*n*, %)	Significance value
AO/OTA classification	Simple	151 (91.5%)	14 (8.5%)	*x*^2^(2) = 3.074, *p* = 0.215
	Wedge	113 (86.3%)	18 (13.7%)	
	Multifragmented	61 (84.7%)	11 (15.3%)	
Gustillo-Anderson classification	Type 1	26 (92.9%)	2 (7.1%)	*x*^2^(2) = 5.657, *p* = 0.059
	Type 2	76 (95.0%)	4 (5.0%)	
	Type 3	223 (85.8%)	37 (14.2%)	
Injury severity score	< 15	248 (88.3%)	33 (11.7%)	*x*^2^(1) = 0.004, *p* = 0.950
	> 15	77 (88.5%)	10 (11.5%)	
Bone loss	No	267 (89.3%)	32 (10.7%)	*x*^2^(1) = 1.492, *p* = 0.222
	Yes	58 (84.1%)	11 (15.9%)	
Head injury	No	293 (87.5%)	42 (12.5%)	Fisher’s exact, *p* = 0.153
	Yes	32 (97.0%)	1 (2.3%)	
Compartment syndrome	No	307 (88.7%)	39 (81.8%)	Fisher’s exact, *p* = 0.301
	Yes	18 (11.3%)	4 (18.2%)	

Management of non-union varied widely between patients and between those with different initial fixation strategies. Infection was implicated in 28% cases of non-union and further management included debridement of infected tissue and antibiotic therapy. After excluding those who were not fit or refused further management of non-union (3 patients), an average of 1.718 subsequent operations were required to achieve successful union, ranging from 1 to 5 additional operations. In 44% of cases, more than one operation was required to address non-union. A list of the operative strategies for addressing non-union can be found in Table 3.

**Table 3:** Description of initialoperative strategy for addressing non-union

Index procedure	Initial treatment of non-union	*n*	Successful, *n*	Successful, %
IM nails (*n* = 16)	Nail dynamisation	6	3	50
	Exchange nail	4	3	75
	Revision to plate	2	2	100
	Revision to frame	1	1	100
	Biological augmentation	2	1	50
	No further ops	1	0	0
Circular frame (*n* = 22)	New/revised frame	11	6	56
	Biological augmentation	5	4	80
	Observation	3	3	100
	LIPUS	2	1	50
	Revision to plate osteosynthesis with biological augmentaion	1	1	100
Plate osteosynthesis (*n* = 3)	Conversion to IM nail	1	1	100
	Conversion to frame	1	1	100
	No further ops	1	0	0
Non operative	No further ops	1	0	0
Modular rail system	LIPUS	1	1	100

*LIPUS* low-intensity pulsed ultrasound

Two patients who sustained grade 3A open tibial fractures following a fall from standing height died shortly after the diagnosis of non-union. One of these patients was a 93-year-old female with a background of dementia who resided in a long term care facility and was initially managed non-operatively. No further treatment was offered to this patient and was subsequently discharged back to a care facility with a full-time lower limb brace. The second patient was a 96-year-old female with a background of osteoporosis and ischaemic heart disease who was initially managed with plate osteosynthesis. This patient was deemed too frail for any further treatment and managed in a full-time lower limb brace. The patient was re-admitted with an ipsilateral femur fracture following a further fall and underwent plate osteosynthesis due to concurrent total knee arthroplasty. Both of these patients died within 6 months of non-union diagnosis.

Two patients who sustained grade-3B open tibia fractures had eventual lower limb amputations after failed lower limb reconstruction. Both patients sustained segmental bone loss of 10 and 30 mm. One patient sustained bilateral grade-3B open tibial fractures which were managed with a modular rail system on one side and Ilizarov frame on the other. Both fractures failed to unite, and these were successfully managed with revision of Ilizarov frame and LIPUS (low-intensity pulsed ultrasound). One patient who was managed with an IMN refused further treatment to progress union and was kept in a Sarmiento brace. Six patients underwent dynamisation of IMN. This was successful in half of the cases and two of these subsequently required exchange nailing to achieve union.

Excluding patients who declined further non-union management or eventual amputation, a total of 39 patients remained, all of whom achieved eventual union. At final follow up 29 (74%) patients were documented to be back at work, social activities or pre-injury baseline function. Three patients developed chronic osteomyelitis requiring further treatment. Four patients developed chronic lower limb pain or chronic regional pain syndrome. One patient developed an ischeamic contracture of the forefoot. This was successfully treated with tendon lengthening and the patient subsequently returned to full pre-injury activities. One patient was left with permanent lymphoedema and one patient was left with a permanent foot drop, however this was a result of the initial injury as opposed to an operative complication.

To illustrate the complexities and bespoke decision making required in the operative management of tibial non-union, we present the following two case examples. In both cases, a multimodal approach was used to augment both the mechanical and biological environment.

### Case 1

**Fig. 1:**
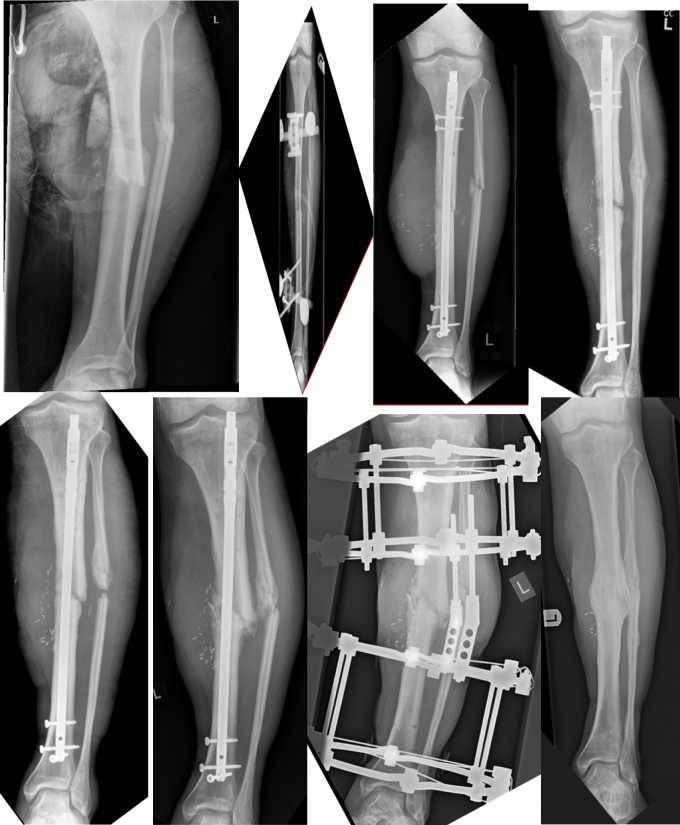
Top left to right: initial injury, temporary external fixator, definitive IMN, subsequent non-union. Bottom left to right: IMN dynamisation, progressive deformity, application of Ilizarov frame and fibular osteotomy, final plain films

A fit and well 45 year old male sustained a grade-3B open left diaphyseal tibia fracture following a RTA. He underwent debridement, external fixation and subsequent definitive IMN with free flap soft tissue coverage. Unfortunately, this resulted in atrophic non-union 9 months following injury. Management consisted of IMN dynamisation and biological augmentation with bone marrow aspirate concentration and platelet rich plasma injected at the site of non-union. This was not sufficient and hypertrophic non-union ensued with evolving varus deformity. This was addressed by fine wire circular frame including a hinge to correct the coronal plane deformity. Union was achieved and the patient returned to full function cycling 80 miles at a time (Fig. 1).

### Case 2

**Fig. 2:**
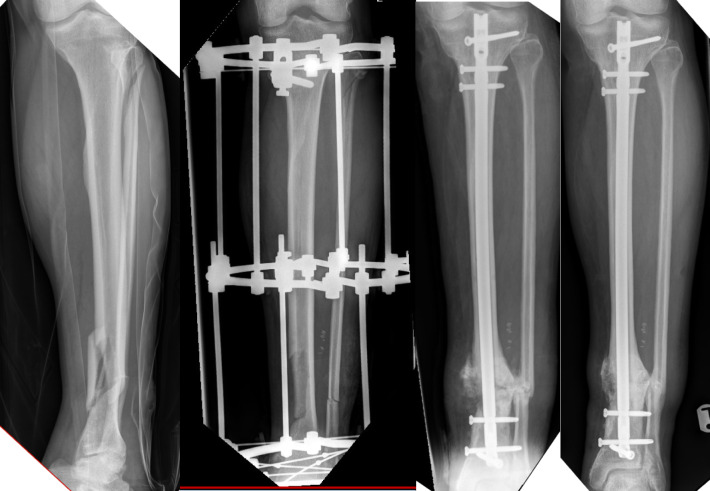
From left to right: initial injury, initial management with Ilizarov frame, acute osteotomy, biological augmentation and antibiotic coated IMN, final plain films

A 50-year-old male with a background of alcohol excess and hepatitis sustained a grade 3A open tibial fracture after a fall from standing height. After initial debridement the patient was left with a non-segmental area of bone loss. The open wound was closed primarily and the fracture was managed in a fine wire circular frame which was removed at 12 months. At subsequent follow up, a progressive varus deformity was apparent and positron emission topography CT using FDG tracer demonstrated an infected non-union. This was managed with debridement, corrective osteotomy and implantation of an antibiotic coated nail. Union was successfully achieved and the patient returned to work and pre-injury function (Fig. 2).

## Discussion

We have presented our experience of diaphyseal tibial non-union and its management. Patient and injury characteristics, operative strategies and eventual outcome have all been described. Diaphyseal tibial non-union occurred at a rate of 11.7% in our cohort. Previous studies report a non-union rate between 1.9 and 7.5% [11–13]. This can be as high as 80% in Type 3 injuries managed by unreamed IMN [11]. The elevated rate in our cohort maybe down to the isolation of purely open diaphyseal tibial fractures. In addition, our institution’s status as a level 1 trauma unit displays an element of selection bias with a large proportion of high energy and multiple injured patients in comparison to other institutions.

There have been several studies attempting to elucidate the risk factors for the development of non-union. These are generally separated into general and local risk factors. General factors shown to play a role include female gender, smoking status and poorly controlled diabetes mellitus amongst others [14]. Local factors relate to the injury and associated soft tissue damage. Open fractures have consistently demonstrated higher rates of non-union than closed injuries. This may be in part due to the higher rate of infection and disruption of the soft tissue envelope and therefore vascular supply of the fracture site. In addition, fracture morphology has also been implicated with unstable multifragmented fractures and those with associated bone loss adding risk. The mechanical environment produced by fixation methods, persistence of fracture gap and the degree of soft tissue stripping required to achieve reduction are the modifiable operative factors that have been incriminated [2, 15–17]. In our cohort, patients who developed non-union following open diaphyseal tibia fractures tended to be older with a higher Charlson comorbidity index score, although these trends did not achieve statistical significance. Similar to previous studies [14, 16], comorbid risk factors included diabetes, peripheral vascular disease, and heart failure of which peripheral vascular disease was a statistically significant risk factor. In addition, smoking status did not appear to correlate with the development of non-union. However, demographics such as smoking status and BMI were incomplete for our cohort. Open tibia fractures that developed non-union tended to be multifragmented, associated with segmental or incomplete bone loss and type-3 open injuries. Patients who developed compartment syndrome following tibial fractures also had a higher rate of non-union compared to those who did not. The association between delayed union and compartment syndrome has been previously described [18] and highlighted as early as 1987 [19]. Therefore, it is unsurprising that the development of compartment syndrome can lead to non-union by the same underlying mechanism of reduced tissue perfusion and damage to the soft tissue envelope. In our cohort, non-union was independent of polytrauma, occurring equally in isolated and multiply injured patients (*p* = 0.950). However, those patients who sustained a concurrent head injury developed non-union less frequently (12.5% vs. 2.3%). Despite this seemingly exhaustive list of risk factors, there is also an evolving suspicion over a genetic predisposition for the development of non-union and owing to the wide variation in union times amongst patient, injury and fixation matched individuals. This is a topic of ongoing and extensive research [20] and may account for the cases of non-union that occur outside of previously documented risk factors.

At our institution management of non-union varied widely between patients with 44% of patients requiring more than one subsequent operation to achieve union. In general, revision fixation with or without biological augmentation appeared to be the most successful strategy and is aligned with the concept of adequate mechanical stability as a cornerstone of management. In the case of patients managed with intramedullary nails, dynamisation had a success rate of 50%, with two patients subsequently requiring exchange nailing. In contrast, those treated by exchange nailing in the first instance had a success rate of 75%. Therefore, it may be more efficient to exchange IMN devices in the first instance, rather than dynamise. Furthermore, a dynamisation procedure may detract from mechanical stability resulting progressive deformity as illustrated by our case example. Patients whose tibial fracture were initially managed by fine wire frame differed from those managed by IMN. As fine wire frames may be modified, there was less incentive for complete revision fixation. It also affords the ability to dial in compression across the fracture site, analogous to nail dynamisation, on an outpatient basis. This accounts for the number of cases where non-union was successfully managed without additional operative procedures.

A number of patients were unable to undergo further management of non-union due to poor physiological reserve or patient refusal. Two patients with infected non-unions and associated bone loss had eventual lower limb amputation. The remaining patients (90%) all achieved eventual fracture union. Several patients returned to full pre-morbid function demonstrating that, although treatment strategies can be prolonged and complex, a successful outcome can be secured for the majority of patients.

There are several limitations to our study. As data was gathered retrospectively, there will be an element of information bias. This is reflected in that not all data points were available for all patients for example BMI and smoking status. Although we have documented complications and return to function, a lack of patient-reported outcome data and objective functional assessment detracts from this.

**Fig. 3:**
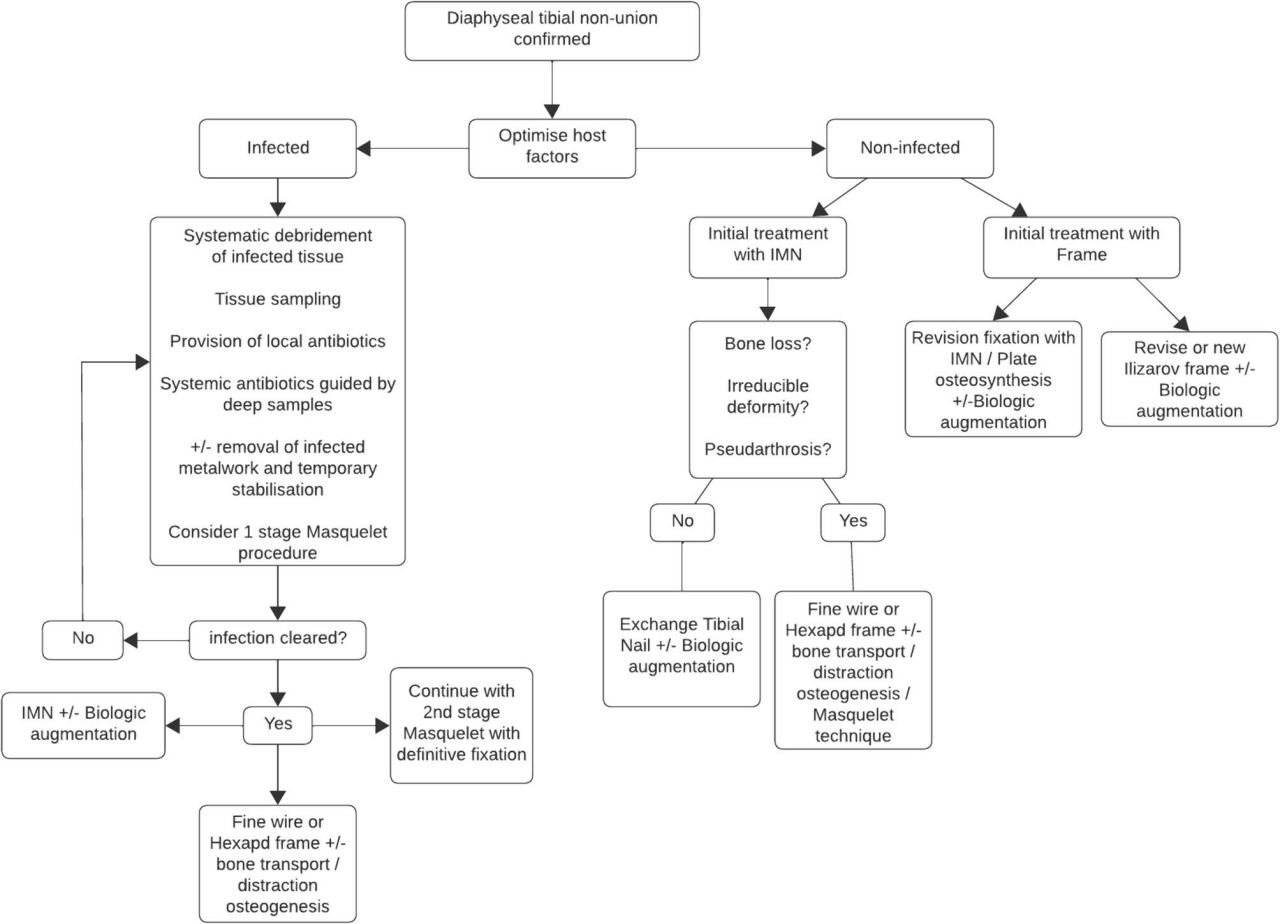
Decision tree for the management of non-union in open tibial shaft fractures

Management of tibial non-union can be complex and convoluted. To overcome this heterogeneity, we propose a simplified decision tree for the management of non-union based on our experience as a high-volume tertiary referral service for fracture non-union (Fig. 3).

## Conclusions

Non-union after open tibial fracture remains a challenging complication which can be associated with long lasting treatment and increased health care costs. Addressing both the biological and mechanical components of the non-union and optimising patient comorbidities remain essential for a successful outcome as seen in this series of patients. We have described our institutional experience of managing diaphyseal tibial non-unions over a 12 year period and demonstrated that a successful outcome can be achieved in the majority of patients. We have included an algorithm to help guide decision making based on this experience.

## Data Availability

Data supporting this study cannot be made available as the participants did not agree to their data being shared publicly.
